# Supplementary material for: Effects of tetracycline on myocardial infarct size in obese rats with chemically-induced colitis
Source: PLoS One. 2019 Nov 12;14(11):e0225185. doi: 10.1371/journal.pone.0225185 (PMC6850547; doi:10.1371/journal.pone.0225185)
Supplement: S1 Table — Data are mean ± SD. Group legends: controls (CON), tetracycline-treated controls (TTC), diet-induced obesity (DIO), diet-induced obesity plus chemically induced colitis (DIO + CIC), tetracycline-treated rats with diet-induced obesity plus chemically induced colitis (DIO + CIC + TTC). (DOCX) [file pone.0225185.s001.docx]

**S1 Table.** The values of heart rate (beats/min) in the experimental groups. Data are mean ± SD. Group legends: controls (CON), tetracycline-treated controls (TTC), diet-induced obesity (DIO), diet-induced obesity plus chemically induced colitis (DIO + CIC), tetracycline-treated rats with diet-induced obesity plus chemically induced colitis (DIO + CIC + TTC).

| Groups | Baseline | Reperfusion | | | | | | |
| --- | --- | --- | --- | --- | --- | --- | --- | --- |
|  |  | 15 min | 30 min | 45 min | 60 min | 75 min | 90 min | 120 min |
| CON | 243 ± 18 | 218 ± 28 | 224 ± 22 | 205 ± 31 | 219 ± 26 | 201 ± 34 | 198 ± 27 | 195 ± 25 |
| TTC | 265 ± 23 | 226 ± 32 | 216 ± 36 | 218 ± 28 | 208 ± 30 | 204 ± 21 | 200 ± 32 | 202 ± 34 |
| DIO | 251 ± 21 | 191 ± 35 | 203 ± 32 | 196 ± 38 | 195 ± 33 | 190 ± 24 | 189 ± 37 | 192 ± 33 |
| DIO+CIC | 249 ± 16 | 205 ± 37 | 208 ± 34 | 212 ± 26 | 198 ± 22 | 189 ± 32 | 192 ± 28 | 194 ± 27 |
| DIO+CIC+TTC | 254 ± 25 | 187 ± 39 | 195 ± 35 | 194 ± 29 | 190 ± 27 | 186 ± 34 | 185 ± 38 | 183 ± 35 |
