# Supplementary material for: Effects of tetracycline on myocardial infarct size in obese rats with chemically-induced colitis
Source: PLoS One. 2019 Nov 12;14(11):e0225185. doi: 10.1371/journal.pone.0225185 (PMC6850547; doi:10.1371/journal.pone.0225185)
Supplement: Attachment — Submitted filename: Response to the comments by Reviewers.docx [file pone.0225185.s003.docx]

**Response to the comments by Reviewer 1**

We would like to thank the reviewer for the constructive comments on the manuscript.

1. **Critique**: “Majority comments answered, failed to update text in terms of recent changes of microbiome in context of obesity which is common comorbidity in patients, add this specific references suggested in original first critiques/comments.”

**Response**: We have modified the discussion (p. 21) and added the relevant reference according to the reviewer’s advice.

**Response to the comments by Reviewer 2**

We would like to thank the reviewer for the fair criticism of the manuscript.

1. **Critique**: “Please address in the discussion section that the changes in myocardial infarct size were not reflected by changes in performance of the isolated heart subjected to ischemia-reperfusion.”

**Response**: This issue is now discussed in the Discussion section (please see changes in p. 17).
